# The Ontario Mother and Infant Study (TOMIS) III: A multi-site cohort study of the impact of delivery method on health, service use, and costs of care in the first postpartum year

**DOI:** 10.1186/1471-2393-9-16

**Published:** 2009-04-28

**Authors:** Wendy Sword, Susan Watt, Paul Krueger, Lehana Thabane, Christine Kurtz Landy, Dan Farine, Marilyn Swinton

**Affiliations:** 1 School of Nursing, Faculty of Health Sciences, McMaster University, 1200 Main Street West, Hamilton, Ontario, Canada L8N 3Z5; 2 School of Social Work, McMaster University, 1280 Main Street West, Hamilton, Ontario, L8N 4M4, Canada; 3 Department of Clinical Epidemiology & Biostatistics, Faculty of Health Sciences, McMaster University, 1200 Main Street West, Hamilton, Ontario, L8N 3Z5, Canada; 4 St. Joseph's Health System Research Network, 99 Wayne Gretzky Parkway, Suite 105, Brantford, Ontario, N3S 6T6, Canada; 5 Biostatistics Unit, St Joseph's Healthcare, 50 Charlton Avenue East, Hamilton, Ontario, L8N 4A6, Canada; 6 Department of Obstetrics and Gynaecology, Mount Sinai Hospital and University of Toronto, 600 University Avenue, Toronto, Ontario, M5G 1X5, Canada

## Abstract

**Background:**

The caesarean section rate continues to rise globally. A caesarean section is inarguably the preferred method of delivery when there is good evidence that a vaginal delivery may unduly risk the health of a woman or her infant. Any decisions about delivery method in the absence of clear medical indication should be based on knowledge of outcomes associated with different childbirth methods. However, there is lack of sold evidence of the short-term and long-term risks and benefits of a planned caesarean delivery compared to a planned vaginal delivery. It also is important to consider the economic aspects of caesarean sections, but very little attention has been given to health care system costs that take into account services used by women for themselves and their infants following hospital discharge.

**Methods and design:**

The Ontario Mother and Infant Study III is a prospective cohort study to examine relationships between method of delivery and maternal and infant health, service utilization, and cost of care at three time points during the year following postpartum hospital discharge. Over 2500 women were recruited from 11 hospitals across the province of Ontario, Canada, with data collection occurring between April 2006 and October 2008. Participants completed a self-report questionnaire in hospital and structured telephone interviews at 6 weeks, 6 months, and 12 months after discharge. Data will be analyzed using generalized estimating equation, a special generalized linear models technique. A qualitative descriptive component supplements the survey approach, with the goal of assisting in interpretation of data and providing explanations for trends in the findings.

**Discussion:**

The findings can be incorporated into patient counselling and discussions about the advantages and disadvantages of different delivery methods, potentially leading to changes in preferences and practices. In addition, the findings will be useful to hospital- and community-based postpartum care providers, managers, and administrators in guiding risk assessment and early intervention strategies. Finally, the research findings can provide the basis for policy modification and implementation strategies to improve outcomes and reduce costs of care.

## Background

The caesarean section rate in Canada, other developed countries, and most regions of the developing world continues to rise [1-5]. For example, approximately 26% of babies born in Canadian hospitals were delivered by caesarean section in 2006 compared to 23% in 2001 and 17% in 1993 [6]. This trend is attributable, in part, to both changes in maternal characteristics and changes in obstetrical practices [7]. Studies conducted in developed countries have found that older maternal age and pre-pregnancy obesity, both growing societal trends, are associated with an increased likelihood of having a caesarean section [3,8-10]. Health service factors, such as the increased use of antenatal care services and births in private institutions where the prevalence of caesarean births is higher than in public settings, play a role in rising caesarean section rates in developing countries [4]. In many centres worldwide most infants in breech position are now delivered by caesarean section, which is largely due to the finding of the Term Breech Trial that caesarean section is safer than vaginal breech delivery [11-13].

A caesarean section is inarguably the preferred method of delivery when there is good evidence that a vaginal delivery is risky to the health of a woman or her infant. However, concerns have been raised over the high caesarean birth rates that far exceed the World Health Organization's (WHO) recommended maximum rate of 15% [14]. Discussions of reasons for the rise in caesarean section rates have centred on the changing attitudes of care providers and women [2]. A substantial number of obstetricians are proponents of caesarean delivery without medical indication [1,15,16]. It has been suggested that a lowering of the threshold for deciding to perform a caesarean delivery and fear of litigation for not performing a caesarean section are main factors contributing to rising rates [1,17,18]. While maternal request for caesarean section also is a factor, due to lack of data the actual number of women requesting caesarean delivery in the absence of clear medical indication and its contribution to rising rates are unknown [19].

Decisions about caesarean section when there is no clear clinically important reason should be informed by a variety of evidence. Caesarean section is a relatively safe procedure, yet there are documented risks for mother and baby including complications of anesthesia and surgery, respiratory distress, and longer term reproductive consequences [18,20]. Maternal morbidity increases with each caesarean section, which is attributable mostly to placenta accreta or the need for hysterectomy [21]. On the other hand, some women experience significant pelvic floor trauma as a consequence of vaginal childbirth [22]. In particular, operative vaginal delivery, especially the use of forceps, increases risk for anal sphincter injury [23]. While knowledge of risks and benefits of different childbirth methods is essential for informed decision making, there is lack of sold evidence of the short-term and long-term risks and benefits of a planned caesarean delivery compared to a planned vaginal delivery [19,24,25]. For instance, study findings regarding the effect of vaginal birth compared to caesarean section on urinary incontinence and postpartum depression often are unclear [26,27], and there is very little published research on the association between delivery method and other outcomes such as breastfeeding duration and functional health status.

It also is important to consider the economic aspects of delivery method. Hospital costs generally are higher for a caesarean delivery than for vaginal delivery, with instrumental vaginal delivery costs being higher than those for spontaneous vaginal delivery [6,28,29]. In addition, women who have a caesarean section or operative vaginal delivery are more likely to be re-hospitalized for complications such as puerperal infection, wound problems, and thromboembolic conditions [30,31]. Very little attention has been given to longer term health care system costs that take into account services used by women for themselves and their infants following hospital discharge, which has implications for the real costs of caesarean section and operative vaginal delivery. A single published study conducted in Scotland that addressed post-discharge costs found no significant differences in cost of community care related to delivery method [29].

The Ontario Mother and Infant Study (TOMIS) III will add to the body of knowledge regarding relationships between mode of delivery method and health outcomes by following a large cohort of women and examining a comprehensive range of physical and psychosocial health indicators over the course of the first postpartum year. This approach is in contrast to previous studies that generally have addressed a specific postpartum health issue over the short-term and usually in a single hospital or clinical practice setting. TOMIS III also will address the knowledge gap related to postpartum service use and cost of care over the long term. It will investigate, in the context of a universal health care system, health services utilization by mothers and infants following hospital discharge and relationships to delivery method.

## Study purpose and research questions

The purpose of TOMIS III is to examine relationships between method of delivery and maternal and infant health, service utilization, and cost of care at three time points during the year following postpartum hospital discharge (6 weeks, 6 months, and 12 months). The primary research questions are as follows:

1. Does method of delivery (vaginal vs. caesarean section) affect rates of postpartum depression at 6 weeks following hospital discharge?

2. Does method of delivery (vaginal vs. caesarean section) affect maternal health at 6 weeks following hospital discharge?

3. Does method of delivery (vaginal vs. caesarean section) affect infant health at 6 weeks following hospital discharge?

Postpartum depression was chosen as the main outcome of interest because the mental health status of childbearing women is a major public health issue [32]. Some research has been found a relationship between delivery method and postpartum depression [33] but a significant association has not been established [26]. Postpartum depression has been shown to affect service use and costs of care [32,34]. Other maternal health outcomes include self-reported health status, functional health status, physical health problems, pelvic floor trauma, and breastfeeding.

Secondary research questions are:

1. Does method of delivery (vaginal vs. caesarean section) affect rates of postpartum depression, maternal health, and infant health at 6 months and 1 year following hospital discharge?

2. Does method of delivery (vaginal vs. caesarean section) affect service use and costs of care at 6 weeks, 6 months and 1 year following hospital discharge?

3. What are the best predictors of postpartum depression, maternal health, and infant health at 6 weeks, 6 months and 1 year following hospital discharge?

4. What are the best predictors of maternal and infant service utilization and costs of care (e.g., physician visits, ER visits, public health nurse visits) at 6 weeks, 6 months and 1 year following hospital discharge?

5. Are there differences in health outcomes, service use, and costs of care by the specific nature of the vaginal and caesarean section delivery (i.e., unassisted vaginal delivery vs. assisted with forceps or vacuum extraction and Caesarean section planned before labour unplanned before labour)?

6. Are there differences in health outcomes, service use, and costs of care by whether the delivery was an intended vaginal delivery or an intended caesarean section?

7. What are postpartum women's perspectives regarding the findings related to patterns of postpartum health and service use?

8. What are the health care providers' perspectives regarding the findings related to site-specific patterns of postpartum health and service use?

The question related to differences by intended delivery method is important in light of the debate regarding the benefits of caesarean section over vaginal delivery. As noted by Hannah [35], a planned vaginal birth may result in an emergency caesarean section, which carries greater risk for the mother than an elective caesarean section, and a vaginal birth may require forceps delivery or result in tearing of the anal sphincter, thus increasing the risk of urinary and fecal incontinence, negative outcomes that are avoided with a caesarean section. It is not known whether a planned caesarean section is more beneficial or harmful than a planned vaginal delivery.

## Methods and design

The design of TOMIS III was based on two previous studies of postpartum health and service use. TOMIS was conducted in 1998–2000 in response to an identified lack of information about health outcomes and service utilization at a time when a postpartum hospital stay of ≤48 hours following uncomplicated vaginal delivery had become standard practice in many jurisdictions [36]. TOMIS II, conducted in 2001–2003, was initiated following implementation of the universal Hospital Stay and Postpartum Home Visiting Program in Ontario and focused on comparison of data pre- and post-program implementation [37]. In both studies, women who had given vaginal birth to a healthy infant were recruited from five Ontario hospitals and followed to 4 weeks post hospital discharge.

TOMIS III is a multi-site study in which a quantitatively-driven sequential mixed methods design was applied. A type of prospective cohort study, a panel study, was used to survey postpartum women regarding delivery method and a variety of health and service use outcomes. A qualitative descriptive method was used to supplement the survey approach, with the goal of assisting in interpretation of data and providing explanations for trends in the findings [38].

## Ethical considerations

The study was approved by the Hamilton Health Sciences/McMaster University Faculty of Health Sciences Research Ethics Board and by the research ethics board of each participating hospital. The following considerations were addressed: participation was voluntary; refusal to participate did not affect care received; participants were fully informed of the nature of the study and of their rights and obligations; signed, informed consent was obtained from all participants; and participants were assured that their information would be kept confidential.

Procedures were developed to protect the confidentiality of data collected for the study in accordance with research ethics boards' requirements and Canadian privacy legislation. These procedures included: training all interviewers and research assistants on confidentiality and having them sign a confidentiality statement agreeing to adhere to study procedures to safeguard data; assigning of study numbers and separation of names and contact information from the data; storage of questionnaires in locked cabinets in locked offices, accessible only to the research team; and storage of electronic data on password protected computers kept in locked offices.

## Prospective cohort panel study

This investigation employed a panel study, a type of prospective cohort study, to collect information from the same participants regarding their exposures and outcomes at multiple points in time [39]. The main *independent variable *of interest in this study is method of delivery. However, evidence suggests that in addition to delivery method, pre-existing health problems [40-42], demographic and psychosocial characteristics [33,43,44], employment status [43], satisfaction with the delivery experience [45], readiness for discharge [46], and access to services [47] can influence maternal and/or infant health. These same factors along with satisfaction with health services can influence service use [42,48,49]. Other *independent variables *of interest include hospital site, parity, age, marital status, income, education, ethnicity, language, immigrant versus non-immigrant, subjective social status, employment status, type of care provider(s), readiness for discharge, maternal concerns at time of discharge, mother's learning needs post-discharge, perceived adequacy of available help/support at home, social support, and satisfaction with services. The main *dependent variables *of interest are postpartum depression, self-perceived health status, health-related functional status, and infant health status. Other *dependent variables *of interest are maternal physical health problems (including pelvic floor trauma), infant health problems, maternal and infant readmission, breastfeeding initiation and continuation, post-discharge health service utilization, and costs of care.

### Sample

Our aim was to recruit women from two hospitals in each of the five geographic regions of Ontario as defined by the Ontario Hospital Association [50]. These hospitals were selected based on their classifications as defined in Women's Health – An Excerpt of the Hospital Report 2002 [51] and the Family-Centred Maternity and Newborn Care National Guidelines [52]. Acute hospitals are classified as small, community or teaching, and a Level III facility is distinguished by its ability to care for women with pregnancies that may be at risk and for infants with severe illness, including a neonatal transfer program. Our aim was to select one Level I or Level II community hospital and one Level III teaching hospital in each region. The North region was an exception where, due to a small number of annual births at most community hospitals, we selected the two largest community hospitals that both became Level II teaching hospitals effective September 2005.

The inclusion criteria for participants in the study were as follows: woman of ≥16 years of age; delivery of a live singleton infant; gestational age ≥37 weeks; mother assuming care of infant when discharged; mother competent to give consent; and mother can be contacted by telephone. Women were ineligible to participate if their infant required admission to a neonatal intensive care or special care nursery for more than 24 hours or were unable to communicate in one of the four study languages (English, French, Chinese, Spanish).

### Sample Size Calculation

In determining the sample size, we assumed equal participation rates between women with a vaginal delivery and women with a caesarean section delivery. Parity was accounted for in the sample size calculation. The goal is to test the null hypothesis that the proportion of depression is identical in the two groups. Postpartum depression was treated as a binary outcome. The criterion for significance was set at alpha = 0.05. The test was two-tailed and the power was set at 80% to detect a minimal clinically important difference of 5% between the two groups (i.e., 10 vs. 15%). A difference of less than 5% would not be of clinical or substantive significance. It also was a reasonable difference that could be expected in this type of research. These assumptions led to a sample size of 690 per group (i.e., 1380 in both groups), and this also would provide 95% confidence intervals of the difference between the percentages to be within plus or minus 3 percentage points.

These calculations were performed using Power and Precision™ software [53]. The overall sample was inflated to account for the expected intra-class correlation (ICC) structure within a hospital [54]. The calculations were based on ICC of 0.018 from previous studies. We selected to use a sample of 3774, which corresponded to an average cluster size of n = 50. It was adjusted by adding at least three respondents for each degree of freedom for all predictor variables and accounted for a potential 30% attrition rate. This assumed rate was based on the results of TOMIS and TOMIS II. A quota (proportionate) sampling strategy was used to determine the number of participants from each hospital site.

We expected the sample size to remain fairly constant after initial attrition because women who agreed to participate in the study but could not be reached for the 6-week or 6-month follow-up were contacted again at the next data collection point. We also used a number of strategies to optimize retention in the study, including asking women to provide telephone numbers of two individuals who would know how to contact them in the case of a move and/or telephone number change. In addition, we kept in touch with women during the year they were enrolled in the study by mailing them a letter with a study magnet at 4 weeks post-discharge thanking them for agreeing to participate in the research and sending them study newsletters at 5 and 10 months.

### Recruitment and Consent Process

Each hospital identified a site project manager, who was a member of the care team for the postpartum unit, to coordinate recruitment and data collection. These site managers received an in-person orientation to the study by the research coordinator who also trained them in subject recruitment, data collection, and use of a tracking form to determine eligibility and participation rates.

Study posters were displayed in relevant outpatient and inpatient hospital settings to alert potential participants that they might be approached to take part in the study. Postpartum unit nurses or site managers attempted to assess all women for eligibility during the recruitment period and to invite all eligible women to participate. The number of women not assessed at each hospital site was documented. Women who were assessed and eligible received a study information letter. Signed informed consent was obtained from those who agreed to participate and women were given a self-administered questionnaire to complete prior to hospital discharge.

### Quantitative Data Collection

Data collection began in April 2006 and ended in October 2008. The data collection tools used in TOMIS [36] and TOMIS II [37] were used in TOMIS III with some modifications. These instruments included a self-administered Mother's Questionnaire completed in hospital and a structured telephone interview schedule post discharge. Both instruments were pilot tested to ensure that skip patterns were clear and to determine if interviewer gender needed to be considered. All study materials and instruments were available in four languages: English, French, Spanish and Chinese.

The Mother's Questionnaire included questions about: baby's birth date, gender and birth weight; type of feeding; infant health problems; pregnancy complications; infant feeding; infant health; obstetrical history; chronic health problems; pregnancy complications; medical problems post delivery; care providers; rating of prenatal services in the community; type of delivery; reasons for caesarean section; length of labour; perceptions of adequacy of help and support at home; readiness for discharge; concerns related to self and infant; and sociodemographics similar to those collected in the National Population Health Survey [55]. The sociodemographic information captured birth date; language spoken at home; self-identified ethnic or cultural group; place of birth and if born abroad, length of time in Canada; marital status; number of other children at home; family income; highest level of education; and the MacArthur Scale of Subjective Social Status, using both the SES ladder and community ladder [56].

Site project managers extracted data from the labour and delivery record, specifically, information about gestation, the nature of the delivery (e.g., use of forceps/vacuum extraction), integrity of the perineum, anesthesia, excessive bleeding, APGAR scores, and health care professionals present at the delivery.

At 6 weeks, 6 months, and 12 months after hospital discharge, trained interviewers contacted study participants by phone. The interviewers were female as women involved in pilot testing of the instruments indicated a strong preference for same gender interviewers. All of the interviewers spoke English, and some were fluent in French, Spanish, Mandarin or Cantonese. Up to ten attempts at various times of the day and days of the week were made to contact study participants. For those unable to complete the interview at the time of the call, a more convenient time was arranged. The 6-week interview schedule was modified slightly for the 6-month and 12-month interviews (e.g., deleting questions regarding length of stay, changing the stem of questions to reflect the appropriate data collection point).

The interview schedule incorporated the Edinburgh Postnatal Depression Scale (EPDS) [57]. The 10-item EPDS has been widely used as a measure of postpartum depression; it is not a diagnostic tool but rather an internationally used screening and research tool. Its psychometric properties are as follow: sensitivity 86%, specificity 78%, positive predictive value 73%, split-half reliability 0.88, and alpha coefficient 0.87 [58]. It has been translated into various languages and tested in diverse cultures [59].

The 12-Item Short-Form Health Survey (SF-12) also was included in the interview schedule. The SF-12 was developed from the SF-36; it has established validity and reliability and population norms to use in interpreting scores [60]. This generic measure captures eight dimensions of health-related functional status (physical functioning, role physical, role emotional, mental health, bodily pain, general health, vitality, social functioning) and yields scores for two summary measures: physical component summary and mental component summary. The SF-12 has a multiple R^2 ^of 0.911 in the prediction of the physical component summary of the SF-36 and 0.918 in the prediction of the mental component summary of the SF-36; test-retest correlations at 2 weeks were 0.89 and 0.76 for the scale components [60]. The SF-12 has been used with postpartum populations [61,62] and has been translated into a number of languages [60].

A single-item question adapted from the SF-12, "In general, would you say that since you delivered this baby your health is excellent, very good, good, fair, poor", was used as a global assessment of maternal health. This type of question is commonly used to measure self-perceived health status. A large body of international research has found it to be associated with specific health problems, health service use, and functional status, indicating psychometric robustness [63]. Women were asked a similarly worded question to assess infant health.

Data on specific physical health problems were obtained by asking women to respond yes/no to a list of problems developed from the literature. This list included: exhaustion or extreme tiredness; frequent headaches or migraines; backache; excessive or prolonged bleeding; sore bottom or genital area; pain on the outside of abdomen or front; pain deep inside abdomen; urinary infection (bladder or kidney); haemorrhoids; mastitis/breast infection; sore nipples; and incision problems. Women also had the opportunity to identify other problems. Questions related to infant feeding addressed type of feeding and, if the woman ever breast fed, whether she was still breast feeding. In the case of breast feeding discontinuation, age of baby at the time breast feeding stopped and reasons for discontinuation were ascertained. If the woman was still breast feeding, she was asked how long she planned to continue.

With regard to pelvic floor outcomes, women were first asked if they experienced any urinary or bowel problems and, if so, whether they were problems that were new or had worsened since their recent delivery. Additional information was gathered from women who identified problems that were new or had reportedly worsened. The severity of urinary problems was captured using Sandvik's Severity Index for Urinary Incontinence, a simple two-question validated index with good test-retest reliability (kappa = 0.69 for question 1 and 0.83 for question 2) [64,65]. The impact of urinary incontinence on daily functioning was measured using the seven-item short-form Incontinence Impact Questionnaire (IIQ-7), which is a valid and reliable measure (total score correlation of 0.97 with long form total score) that is sensitive to change [66]. Questions were adapted from Sandvik's index and used language from the Manchester Health Questionnaire [67] to assess severity of fecal incontinence. Impact of fecal incontinence was measured using the Lifestyle Scale of the Fecal Incontinence Quality of Life Scale [68]. This 10-item scale has demonstrated test/retest reliability (alpha = 0.96), acceptable internal reliability (Cronbach's alpha>0.70), and discriminate and convergent validity [68].

To assess sexual function, study participants were first asked if they had been sexually active in the past 4 weeks and, if so, whether they experienced any problems. Those reporting problems were then asked if these problems were new or worse since their recent delivery. Further information about these problems was obtained using the 3-item pain/discomfort and 3-item satisfaction domains of the Female Sexual Function Index (FSFI), whose scoring system allows for individual domain scores [69]. It was tested using three different groups of women and demonstrated validity, good internal consistency (Cronbach's alpha = 0.82 to 0.94 for the selected domains), and test-retest reliability (r = 0.70 to 0.87 for the selected domains) [69].

The interview schedule also included the modified Duke-UNC Functional Social Support Questionnaire used by the consortium for Longitudinal Studies of Child Abuse and Neglect (LONGSCAN) [70,71]. This 10-item instrument contains three subscales measuring affective, confidant support, and instrumental support. Seven items are from the original questionnaire and represent two subscales (alpha coefficients for confidant support = 0.62, affective support = 0.76); the other three items were developed by LONGSCAN to measure instrumental support [71]. Low scores indicating low social support have been reported to be negatively associated with health care utilization [70].

Information also was gathered on: length of postpartum hospital stay; satisfaction with length of stay; unmet learning needs; hospital readmission; satisfaction with services in labour and delivery, services on the postpartum unit, and postpartum services in the community; self-reported health status prior to the pregnancy; history of depression; and employment status.

To address the research questions regarding service use and costs of care, participants were asked to keep track of health care visits, tests and procedures, medication costs, purchases of supplies/equipment, and direct out-of-pocket costs (e.g., travel expenses) using a modified Ambulatory Health Care Record (AHCR) [72]. These data were collected as part of the telephone interview. The AHCR has been used with diverse populations over different time periods. In a study to evaluate this tool, observed agreement between care recipients' reports on the AHCR and administrative data ranged from moderate for specialist visits (0.85) to perfect (1.00) for physiotherapy visits (kappa = 0.41 to 1.00); observed agreements between self-reports of medication and hospital pharmacy databases were high to very high (0.78 to 0.95), with kappas of 0.55 to 0.64 [72].

### Quantitative Data Analysis

Self-report questionnaire data and 6-week interview data will be cleaned and ready for analysis in May 2009, followed by the 6-month and 12-month data later in the year. Data management was done by the Biostatistics Unit at St Joseph's Healthcare, Hamilton via fax modem and TeleForm software. This ensured proper monitoring of data accuracy, quality, and completeness with routine and comprehensive data entry checks. All analyses will be performed using SPSS 16.0 and SAS. The results will be reported for each group: vaginal delivery and caesarean section. Patient demographics, depression score, maternal health, infant health, service utilization and costs of health care will be summarized using descriptive summary measures, expressed as mean ± standard deviation (SD) or median (minimum-maximum) for continuous variables and number (percent) for categorical variables. All statistical tests will be performed using two-sided tests at the 0.05 level of significance. The Bonferroni method will be used to adjust the level of significance for testing each outcome to keep the overall level at alpha = 0.05. For all models the results will be expressed as estimates of coefficients (or odds ratios for binary outcomes), standard errors, corresponding two-sided 95% confidence intervals and associated p-values. P-values will be reported to four decimal places with values less than 0.0001 reported as <0.0001.

Generalized estimating equations (GEE) will be used for all primary outcomes [73]. The GEE is a special generalized linear models technique for clustered or correlated data. It allows for the specification of a correlation structure between patients within a hospital and produces unbiased estimates under the assumption that missing observations will be missing at random. An amended approach of weighted GEE will be employed if missingness is found not to be random [22,74]. Missing values will be handled using multiple imputation techniques [75]. Goodness-of-fit will be assessed by examining the residuals for model assumptions and chi-squared test of goodness-of-fit. For each research question, potential predictors and confounding variables will be selected *a priori *based on findings from the literature, clinical experience, and investigator hypotheses. We will adjust for site, parity, and other factors that are associated with specific health and service use outcomes in the modelling process. Multicollinearity will be assessed by exploring bivariate Pearson's correlations among independent variables and using statistics such as tolerance statistics or variance inflation factors. Highly correlated variables will not be entered into the GEE analyses. Variables will be included in a model only if they significantly improve the fit of the model, are confounding factors, or for theoretical/clinical reasons. The best predictors of each outcome variable of interest (health and service use) will be those variables that remain in the final GEE models (excluding variables which were forced into the final model that did not statistically improve the fit of the model or were not confounding factors).

A valid and reliable tool developed by Browne, Gafni and Roberts will be used to determine costs of care [76]. This tool consists of two components, resource utilization as captured in the Ambulatory Health Care Record and unit cost. Resources used are multiplied by unit costs to arrive at total costs of care. Unit cost associated with readmission is based on the Ontario hospital interprovincial per diem rates for inpatient services whereas unit cost of emergency room visits is based on an average hospital cost plus emergency room physician unit cost [76]. Unit cost per professional visit is determined from appropriate sources such as the Schedule of Benefits for Physician Services, public health and other agency rates, and usual costs of service (e.g., for chiropractor, social worker). Unit cost of medications will be determined using the Ontario Drug Benefit Formulary and non-prescription drugs databases, and unit cost of diagnostic tests and procedures will be based on fees obtained from the Ontario Health Insurance Schedule of Benefits and Fees [76]. Average costs per person (at 6 weeks, 6 months and 12 months post discharge) will be calculated for women and infants as well as per mother/infant dyad. Specific characteristics of mothers and infants (including method of delivery and specific characteristics of the delivery) will be entered into GEE analyses to determine the impact of these variables on health service cost. The best predictors of total cost will be those variables that remain in the final model (excluding variables which were forced into the final model that did not statistically improve the fit of the model or were not confounding factors). It is expected that the distribution of health care cost will be non-normal. Therefore, the lognormal distribution will be assumed for modeling cost.

## Qualitative descriptive study

Preliminary site-specific findings of the Mother's Questionnaire and the 6-week and 6-month interviews provided the foundation for the qualitative descriptive component of the research. The intent was to obtain women's and health care providers' perspectives of the findings and possible explanations for them considering the local context.

### Sample and Recruitment

Women who participated in the survey and postpartum service providers at each study site were recruited for this phase of the study. Women were informed of the opportunity to participate in a focus group via study newsletters and at the end of the 12 month telephone interview. They were asked to contact the research coordinator if they were interested in taking part in a discussion group about their perceptions of the study findings. Purposeful criterion sampling, which allows for the selection of information-rich and varied cases for in-depth study, was used [77]. The aim was to achieve diversity in characteristics such as delivery method, age, ethnicity, income, and the presence/absence of specific health problems. Women selected as focus group participants were sent letters of invitation informing them of the time and place of the group interviews, with a request to reply to the research coordinator. One week prior to the focus group these women received a follow-up telephone call to determine if they would be attending the focus group. We aimed for a sample of 10 to 12 for each site-specific focus group. Women who participated received a $25 gift certificate in appreciation for their time and contribution. Informed signed consent was obtained prior to data collection.

A purposeful sample of postpartum care providers and program managers also was selected for a second site-specific focus group. Our contact person at each study site was asked to identify people from a variety of disciplines (medicine, nursing, midwifery, and social work) from both the hospital and community sectors as potential study participants. These individuals received a letter inviting them to participate in a group interview and were called 2 weeks before the focus group to confirm participation. Again, the target sample was 10 to 12 participants at each site and informed signed consent was obtained prior to participation.

### Qualitative Data Collection

Focus group interviews were used to gather the qualitative data, and were conducted between March 2008 and November 2008. As noted by Johnson and Turner [78], focus groups are appropriate in sequential mixed methods studies "to help researchers better understand and interpret information and findings resulting from the earlier use of other data collection methods" (p. 309). A member of the research team presented site-specific study findings and engaged focus group participants in discussion to capture explanations for the findings based on their personal postpartum health and care experiences and characteristics of local service delivery. The audio from the focus group interviews was digitally recorded.

### Qualitative Data Analysis

The transcribed focus group interviews will be entered into and analyzed with the assistance of NVivo 7.0 qualitative analysis software. Content analysis as recommended for qualitative descriptive studies will be used [79]. Two research team members will review each transcript and assign codes to meaningful units of data (e.g., sentences, phrases), and then compare and reach consensus on coding. These codes will capture perceptions and potential explanations of the quantitative findings. During the course of data analysis, related codes will be subsumed under broader emergent themes and additional codes and categories will be developed as necessary to depict new meanings in the data. The research coordinator will review all transcripts to ensure that the coding scheme has been applied consistently to all data. Excerpts from the transcripts will be used to illustrate themes and categories.

## Discussion

### Operational Issues and Study Protocol Changes

During the course of this study, a number of operational challenges were encountered. While five of the original 10 hospital sites were able to reach their quota sample for the study, the other hospitals experienced recruitment difficulties and did not reach their quota. Challenges varied across the hospitals sites and included limited staff resources, other concurrent research studies being conducted on the unit, participant burden associated with taking part in a longitudinal study, competing demands on the unit such as education for new equipment and accreditation requirements, construction of a new unit, and high staff turnover at both the bedside and administrative levels. One of the hospitals initially chosen as a TOMIS III site was unable to continue after recruiting only 34 women for the study, so another hospital in the same city was added as the 11^th ^study site. The study timeline was negatively affected by these various challenges.

In spite of the difficulties we encountered, 2560 women were recruited into the study. This number represents 68% of our target sample of 3774, which assumed a 30% attrition rate over the course of the study. Six-week data were gathered from 74% of women recruited from all hospital sites (n = 1897). It became impossible to conduct the 6-month and 12-month follow-up with all study participants due to financial constraints. We collected 6-month data from women from nine hospital sites (n = 1823) and 12-month data from women from eight sites (n = 1310), representing 71% and 51% respectively of our initial sample. The disruption in the timeline also precluded conducting focus groups at all study sites; we held focus groups in 7 of the 10 participating communities.

### Study Strengths and Limitations

One of the study's strengths is the use of a prospective cohort design. In contrast to a series of cross-sectional studies that would recruit new participants for each study, this panel study collected information from the same people over time, thereby allowing for measurement of changes in outcomes at the individual level and to relate changes in one variable to changes in another variable. Also, more confidence can be placed in the accuracy of the data collected because participants were not required to recall events for long periods of time and kept a record of health service use and associated costs. Another advantage of the study design is the ability to provide information about temporal relationships, which can be helpful when making statements about the direction of relationships and in hypothesizing possible causal relationships

Other strengths of the study are the inclusion of women from both community and teaching hospitals across the province of Ontario and the inclusion of women who spoke languages other than English. In addition to Canada's other official language, French, the study materials and interviews were available in Chinese (Cantonese and Mandarin) and Spanish. These languages were chosen as they were the other most prevalent languages spoken at home by TOMIS and TOMIS II participants.

The main limitations of the study are related to sampling. The use of a volunteer sample introduces a potential selection bias, especially when participants were expected to be involved in the study over an extended period of time. For instance, women with severe physical or mental health problems or social risk factors may have chosen not to participate or dropped out of the study. However, we will be able to compare demographic characteristics and outcome variables reported in the Mother's Questionnaire between participants that dropped out of the study and those who completed follow-up at the different time points to determine any differences. Another study limitation is that women who could not communicate in one of the four study languages were excluded as were women with seriously ill infants, that is, infants admitted to a NICU or special care nursery for more than 24 hours. However, this latter group represents a very small proportion of the postpartum population and approaching them to participate shortly after giving birth would have imposed an unnecessary burden on them.

Other potential limitations are related to the study design. Attrition can be problematic in panel studies as those who drop out may differ from those who continue to participate. In addition, participants' responses can be influenced by asking the same questions over time and these repeated measures could influence behaviour, thereby biasing study findings. Also, the reliance on self-report data may be viewed as a study limitation. Use of self-report data in the study was dictated, in part, by the extreme difficulty in gaining access to the OHIP database and in linking OHIP data to specific individuals given federal privacy legislation. However, use of secondary data sources for health services research has been criticized due to problems with miscoding of data, missing data, and coding of services or procedures in clinically imprecise ways [80]. Moreover, use of the OHIP database would have captured use of only those services that are billed to the government. For the purposes of the study it was important to capture the full range of health services used by women in the first postpartum year, associated costs (e.g., travel), and out-of-pocket costs for medications and medical supplies and equipment. These data provide a more comprehensive understanding of costs.

### Study Relevance

This study is a prospective examination of maternal and infant health outcomes and relationships to method of delivery. It also addresses whether a planned caesarean delivery is more beneficial than harmful to a woman and her infant compared to a planned vaginal delivery, with analysis based on intended rather than on actual method of delivery. Unlike studies that have focused primarily on specific outcomes (e.g., readmission, pelvic floor trauma, breastfeeding) and often in isolation, this study is a comprehensive examination of multiple health indicators, which allows for multivariate analyses. It is unique in that it examines health service utilization and concomitant costs of care after hospital discharge for women with caesarean section delivery, and includes a comparison with service use and costs for women following vaginal delivery. A sub-sample of women was followed for 1 year, which will provide information about the long-term health consequences and costs and allow examination of changes over time.

The research is especially important given increasing caesarean section rates and the need for more evidence regarding health outcomes and service use associated with delivery method, particularly in light of the increasing emphasis on the use of evidence by both health care providers and patients in making informed choices. The findings can be incorporated into patient counselling and discussions about the advantages and disadvantages of different delivery methods, potentially leading to changes in women's preferences and clinical practices. In addition, the findings will be useful to hospital- and community-based postpartum care providers, managers, and administrators in guiding risk assessment and early intervention strategies. Finally, the research findings can provide the basis for policy modification and implementation strategies to improve outcomes and reduce costs of care, with resource allocation based on population needs.

## Competing interests

The authors declare that they have no competing interests.

## Authors' contributions

WS wrote the grant application, has overall project responsibility, provides overall study direction, and drafted and revised the manuscript. SW, PK, LT, CKL, and DF contributed to the grant application and study design, and provide ongoing direction to the project. MS contributed to revisions to the study design and protocol, and drafted sections of the manuscript. LT is responsible for the statistical and analytic aspects of the study. All authors assisted in editing draft manuscripts, and read and approved the final manuscript.

## Pre-publication history

The pre-publication history for this paper can be accessed here:


